# Viscoelastic Properties of Zona Pellucida of Oocytes Characterized by Transient Electrical Impedance Spectroscopy

**DOI:** 10.3390/bios13040442

**Published:** 2023-03-30

**Authors:** Danyil Azarkh, Yuan Cao, Julia Floehr, Uwe Schnakenberg

**Affiliations:** 1 Institute of Materials in Electrical Engineering 1, RWTH Aachen University, Sommerfeldstraße 24, 52074 Aachen, Germany; 2 Helmholtz-Institute for Biomedical Engineering, Biointerface Laboratory, RWTH Aachen University, Pauwelsstraße 30, 52074 Aachen, Germany

**Keywords:** zona pellucida, oocyte, Young’s modulus, viscosity, electrical impedance spectroscopy, creep curve, Maxwell model, micropipette aspiration, equivalent electrical circuit, microfluidics

## Abstract

The success rate in vitro fertilization is significantly linked to the quality of the oocytes. The oocyte’s membrane is encapsulated by a shell of gelatinous extracellular matrix, called zona pellucida, which undergoes dynamic changes throughout the reproduction cycle. During the window of highest fertility, the zona pellucida exhibits a softening phase, while it remains rigid during oocyte maturation and again after fertilization. These variations in mechanical properties facilitate or inhibit sperm penetration. Since successful fertilization considerably depends on the state of the zona pellucida, monitoring of the hardening process of the zona pellucida is vital. In this study, we scrutinized two distinct genetic mouse models, namely, fetuin-B wild-type and fetuin-B/ovastacin double deficient with normal and super-soft zona pellucida, respectively. We evaluated the hardening with the help of a microfluidic aspiration-assisted electrical impedance spectroscopy system. An oocyte was trapped by a microhole connected to a microfluidic channel by applying suction pressure. Transient electrical impedance spectra were taken by microelectrodes surrounding the microhole. The time-depending recovery of zona pellucida deflections to equilibrium was used to calculate the Young’s modulus and, for the first time, absolute viscosity values. The values were obtained by fitting the curves with an equivalent mechanical circuit consisting of a network of dashpots and springs. The observer-independent electrical readout in combination with a fitting algorithm for the calculation of the viscoelastic properties demonstrates a step toward a more user-friendly and easy-to-use tool for the characterizing and better understanding of the rheological properties of oocytes.

## 1. Introduction

Assisted reproductive technology (ART) is an established therapeutic technique that is offered to couples suffering from reproduction difficulties or is used for preserving and breeding animals. Two main approaches are utilized: in vitro fertilization (IVF) and intracytoplasmic sperm injection (ICSI). In IVF, sperms are added to the oocyte without mechanical assistance. In contrast, for ICSI, a single sperm is injected by impalement of the oocytes with a micropipette. The latest published world report included the country, regional and global estimates of ART utilization, effectiveness, and safety in 2014. Information on global ART practices, intrauterine insemination, pregnancy, and neonatal outcomes are also included [1]. The success of artificial fertilization depends highly on the oocyte quality [2,3]. Oocytes have a gelatinous layer of the extracellular matrix surrounding the membrane, called the zona pellucida (ZP). The mouse ZP, which becomes soft in the window of the highest fertility, is comprised of three proteins (ZP1–ZP3) [4,5]. Therefore, assessing the mechanical properties of the ZP plays a crucial role in improving the IVF and ICSI fertilization rates.

Mice are often used for genetic ablation and mutation to investigate their functional roles in several different biological processes and diseases. This knowledge can then be transferred to humans. In the field of reproductive biology, over two hundred genetically modified mice with impaired fertility have been described [6,7]. More than 90% of the murine genome sequence can be assigned to corresponding regions of the human genome and 80% of the genes are orthologous [8]. The translation to human reproductive biology is an important aspect of humanized genetic mutations in mice. Basic research can be undertaken in the mouse model without violating ethical principles.

Different tools have been published to determine the hardening of the oocyte’s ZP. All techniques are based on measuring the ZP’s deflection during the application of a known force. In microtactile and indentation setups, a needle or a sphere is locally dented into the cell. With regard to mouse oocytes, Murayama et al. used a vibrating needle whose frequency changed during ZP dentation [5,9]. Sun et al. developed a sophisticated surface micromachined force sensor that deformed the ZP. The resulting dentation depth was proportional to the applied force [10]. For both techniques, sophisticated electromechanical transducers had to be developed. Atomic force microscopy (AFM) as well as nanoindenter tools use stiff cantilever beams instead of needles [11,12,13,14]. The force that is induced by the cantilever is proportional to the beam’s deflection. Cantilever-based techniques have been well-established since 1986 [15]. However, the cantilever’s length and stiffness must be adapted carefully to the material under test. The variable shape of an AFM probe will determine the nature of the force–deformation curve.

In comparison, the ICSI-inspired micropipette aspiration technique is often utilized [16,17,18]. Here, the glass micropipette is not used to inject the sperm into the oocyte, but is used to aspirate a cell surface into the pipette by applying suction pressure to the pipette. The length of the penetration of ZP into the pipette, called aspiration length L, is measured by light microscopy. The aspiration length depends on the applied pressure. [19,20,21,22]. The micropipette aspiration technique suffers from the demanding observer-dependent image processing of video recordings with high-resolution contour extraction to measure the aspiration length of the almost transparent ZP.

With all of these approaches, the Young’s modulus of the ZP, which expresses the elastic property and therefore the hardening, can be calculated from the raw data. However, they are challenging in cell handling and the oocytes can only be characterized serially one by one.

**Figure 1:**
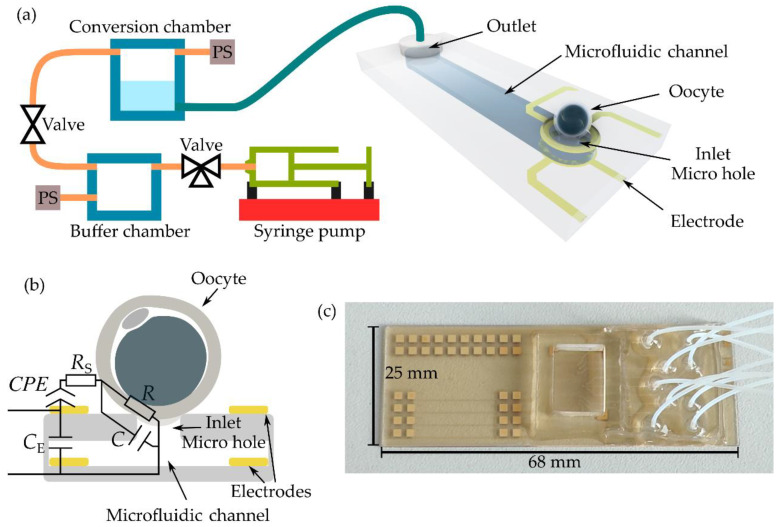
Microfluidic aspiration-assisted electrical impedance spectroscopy system for the characterization of single oocytes. (**a**) Schematic drawing of the setup. PS represents the pressure sensor. (**b**) Schematic cross-section of the electrode arrangement at the microhole combined with the equivalent electrical circuit model. CPE: constant phase element, R_S_: medium resistance, R: resistance of zona pellucida at the rim of the aperture, C: capacitance of zona pellucida and aperture, C_E_: electrode crosstalk capacitance. Drawings in (**a**) and (**b**) are not to scale. (**c**) Photograph of the microfluidic chip. On the left side is the area with 36 contact pads for the wiring of 18 electrodes at nine microholes, in the middle part is the PDMS interposer with the chamber, and on the right side, the area for the tubing connection is shown.

Therefore, we developed a microfluidic approach in which the optical method is replaced with an observer-independent electrical detection method [23,24]. The schematic drawing of the setup is shown in Figure 1a. The microfluidic chip, as the central component of the setup, consists of at least nine microfluidic channels, although only one channel is shown in the schematic. Here, a microhole in the cover lid served as an inlet, whereas the outlet was connected to a tube. Using the manufactured chip depicted in Figure 1c, nine tubes were connected via an interposer to nine conversion chambers of a customized pressure regulation module. In addition, the interposer provides a chamber, filled with cell culture medium before the experiments.

The pressure was defined in a buffer chamber by a programmable syringe pump. This pressure was then applied to the selected conversion chamber via electromagnetic valves. Pressure sensors (PS) were used for pressure control. Using the pressure regulation unit, objects or cells were hydrodynamically trapped in the microholes by applying suction pressure.

Two ring-shaped electrodes were vertically arranged around the microhole, as depicted schematically by the cross-section in Figure 1b. Trapped spheres or cells were characterized by electrical impedance spectroscopy (EIS) [23,24]. EIS is a non-invasive technique that applies a frequency-dependent voltage between the electrodes and measures the corresponding current response to calculate the electrical impedance. To underline the combination of EIS with the microfluidic aspiration technique, we called the setup a “microfluidic aspiration-assisted electrical impedance spectroscopy (MAEIS) system” [24]. Four-point EIS measurements were carried out. The spectra were fitted with the equivalent electrical circuit (EEC) shown in Figure 1c using EIS Spectrum Analyzer software [25]. The model consists of five circuit elements: constant phase element CPE for describing the electrode, medium resistance R_S_, resistance R of the zona pellucida at the rim of the micro hole, the capacitance of zona pellucida C and the microhole, and electrode crosstalk capacitance C_E_, which occurs in the high frequency range. Fitting was conducted at 30 kHz, the frequency of the highest sensitivity [24]. Aside from the impedance magnitude Z, the resistance R was evaluated. R is the only circuit element that depended on pressure. Changes in pressure at the rim of the microhole squeezed the Z,P which led to an increase in resistance.

With applied suction pressure, the ZP aspirated into the microhole. The similarity to the micropipette aspiration technique is obvious. Therefore, a well-established model developed by Alexopoulos et al. was used to calculate the Young’s modulus *E* of the aspirated tissue from the linear dependency of the aspiration length with respect to the suction pressure [26]. *E* can therefore be expressed as

(1) $$E=2C\left(1-v_{\mathrm{ZP}}^{2}\right)\frac{\Delta p}{L/r_{i}},$$

where *υ*_ZP_ is the Poisson ratio of the ZP (*υ*_ZP_ = 0.04 [19]); ∆*p* is the suction pressure; *L* the corresponding length of the aspirated ZP; *r*_i_ is the inner micropipette radius. *C* is a function of the dimensionless ZP shell thickness h* = *h*/*r*_i_ [26] and can be found as Equation (S1) in the Supplementary Materials.

To correlate the aspiration length with the measured impedance, we recently presented the corresponding analytical model [24]. In this approach, it is assumed that the suction pressure dependent impedance increase is correlated to the resistance increase in the ZP at the rim of the microhole. The ZP as an isotropic, homogeneous, elastic, spherical shell with the outer radius *r*_c_ assumed to be fixed at the rim of the microhole and thinned due to stretching into the microhole. The thinning at the rim, and therefore the resistance increase, is represented by the circuit element *R* in the EEC. Depending on the radius *r*_i_ of the microhole, the aspiration length *L* can be correlated with the resistance change ∆*R* in Equation (2) [24]:

(2) $$L=\frac{2\cdot \Delta R/R_{0}}{\frac{R_{2,0}}{R_{0}}\;\left(\frac{1}{r_{C}}+\sqrt{{\left(\frac{1}{r_{C}}\right)}^{2}+\frac{2}{r_{C}\left(r_{C}-\sqrt{r_{C}{}^{2}-r_{i}{}^{2}}\right)}\left(\frac{R_{2}}{R_{2,0}}-1\right)}\right)}$$

where ∆*R* is the change in *R* due to the suction pressure ∆*p*; *R*_0_ is the resistance of the open micro hole without oocyte; *R*_2,0_ is the resistance of the sealed microhole with trapped oocyte at minimum fixation pressure; *R*_2_ is the resistance after recovery of the ZP to equilibrium; *r*_i_ and *r*_c_ are the radii of the microhole and the oocyte, respectively. By substitution of Equation (2) into Equation (1), the Young’s modulus of the ZP can be calculated [24].

In this work, we used the MAEIS setup and applied defined suction pressure steps to the trapped mouse oocytes. In comparison to our previous work [24], transient electrical impedance spectra were recorded and related to time-depending recoveries of ZP deflections to equilibrium after pressure steps. For the first time, these electrically obtained creep curves were evaluated to calculate the mice oocyte ZP’s Young’s moduli and viscosities on the basis of an appropriate equivalent mechanical circuit model consisting of a network of dashpots and springs.

## 2. Materials and Methods

### 2.1. Materials

AZ10-XT and AZ726 MIF were purchased from MicroChemicals GmbH (Ulm, Germany); SUEX K25 dry film photoresist (DFR), SU-8 3050, and mr-Dev600 were from micro resist technology GmbH (Berlin, Germany); Sylgard 184 poly(dimethylsiloxane) (PDMS) and DOWSIL 96-083 silicone adhesive were from DOW Inc. (Midland, MI, USA); NB SEMIPLATE AU 100 TL from NB Technologies GmbH (Bremen, Germany), EmbryoMax Advanced KSOM MR-101-D Embryo medium, (3-aminopropyl) triethoxysilane (APTES), hyaluronidase, acetone, isopropanol, hydrochloric acid, nitric acid, hydrogen peroxide, and ammonium hydroxide solutions for device fabrication were from Sigma-Aldrich Chemie GmbH (Taufkirchen, Germany); oocyte transfer pipettes and mineral oil were from Gynemed (Lensahn, Germany); polytetrafluoroethylene (PTFE) tubes were from Adtech Polymer Engineering Ltd. (Stroud, UK); 6-cm Petri dish from VWR (Darmstadt, Germany); AMS 5812-0050-D-B pressure sensors from Analog Microelectronics GmbH (Mainz, Germany); L7805CV linear voltage regulator from STMicroelectronics N.V. (Plan-les-Ouates, Switzerland); HE721A0500 relay from Littelfuse Inc. (Chicago, IL, USA); LVM105R-5A1U-Q 3-port valve from SMC Corporation (Tokyo, Japan); EZ-Grip and Denudation tips from CooperSurgical Fertility Solutions (Maaloev, Denmark).

### 2.2. Microfluidic Chip Fabrication

The fabrication process of the microfluidic chip was recently published [23,24,27] and is briefly summarized here. Three 25 µm thick SUEX^®^ dry film resist layers were laminated, patterned by standard UV lithography, and in the case of forming the 35–36 µm in diameter microholes, structured by a dry etching process in an inductively coupled plasma reactive ion etching tool (SI 500, SENTECH Instrument GmbH, Berlin, Germany) by applying 60 sccm O_2_, 3 sccm SF_6_, 0.5 Pa, 100 W power, and −150 V DC-bias voltage. The sidewalls of the exposed microholes showed an inclination angle of 86°. The intermediate electrodes, wires, and connection pads were fabricated by the deposition of thin-film seed layers (titanium and gold), standard UV lithography, and gold electroplating. Each electrode was connected by two wires to enable four-point EIS measurements. On top of the chip, a replica-molded poly(dimethylsiloxane) (PDMS) interposer was bonded to the chip by applying oxygen plasma in combination with the deposition of APTES.

### 2.3. MAEIS Setup Assembly

As described in [24], the microfluidic chip was electrically connected to a custom printed circuit board (PCB). The four electrodes for one EIS measurement were manually addressed via mini dual in-line package (DIP) switches. The PCB board was placed under an inverted microscope with a hotplate. The temperature of the medium inside the PDMS frame was kept at 37 °C, and controlled by a PECON TempController 2000-1 (PeCon GmbH, Erbach, Germany) in combination with a Pt100 thermocouple, which was inserted into the medium.

Four-point EIS measurements were carried out between 1 kHz and 10 MHz in 80 logarithmic steps with a 25 mV oscillation voltage using an Agilent 4294A precision impedance analyzer (Agilent Technologies Inc., Santa Clara, CA, USA). A custom LabVIEW (National Instruments Inc., Austin, TX, USA) program was used to control the EIS measurements.

### 2.4. Oocyte Preparation and Handling

Oocytes of two different genetic mouse models with normal and super-soft zona pellucida were used. Fetuin-B wild type mice (WT with C56BL/6 background) showed normal ZP, while fetuin-B/ovastacin double deficient (DKO with the FVB and C57BL/6 mixed genetic background [28,29]) always had a super-soft ZP. All animal experiments were in accordance with the German Animal Welfare law and approved by the State Governments in North Rhine Westphalia, Germany. Maintenance, handling, and treatment of the mice were performed according to the Federation for Laboratory Animal Science Associations recommendations. Mice were hormonally stimulated for the collection of the oocytes in the MII stage. The oocytes were separated, as previously described [11,28]. After isolation, the oocytes were transferred to the microfluidic chip for EIS measurements.

Just before the oocyte was trapped at the microhole, an EIS spectrum was taken as the blank control. From this reference spectrum, the fitted resistance R at the frequency of highest sensitivity, defined as the resistance *R*_0_ of the open microhole, was used for the calculations. For the measurements, an oocyte with the diameter of around 100 µm was positioned near the microhole with a micropipette. After applying suction pressure to the microchannel, the oocyte was trapped hydrodynamically at the microhole. The trapped cell provided a tight seal of the microhole and stayed in the trapped position when a minimum suction pressure of 1 hPa was applied. In the following, the minimum fixation pressure is defined as 0 hPa as a reference with regard to the applied suction pressure. EIS measurements were carried out while the suction pressure was subsequently increased from 1 reference pressure to 7 hPa in 1 hPa incremental steps.

## 3. Results

Transient EIS measurements were carried out on four wild-type (WT) and four fetuin-B ovastacin double deficient (DKO) MII oocytes. Each measurement included a set of seven creep curves related to seven incremental pressure steps.

**Figure 2:**
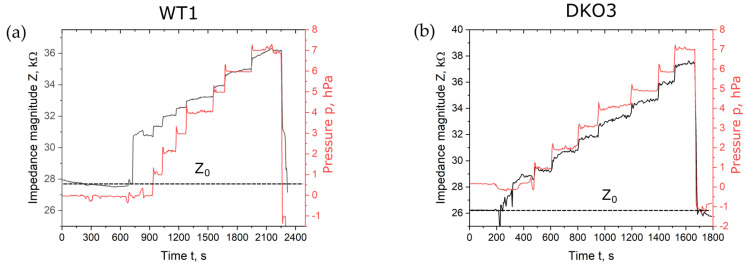
Impedance magnitude with regard to time of one measurement with seven suction pressure steps of 1 hPa for oocyte WT1 (**a**) and DKO3 (**b**). Impedance measurements were carried out at a frequency of 30 kHz. *Z*_0_ shows the blank impedance magnitude value of the open microhole without the trapped oocyte.

Figure 2 shows the magnitude of impedance over the time span of the seven incremental suction pressure steps of 1 hPa, displayed representatively for oocytes WT1 and DKO3. The measurement started with an open microhole. The first jump in impedance magnitude was correlated with the trapping of the oocyte in the microhole. The pressure overshoot at the beginning of each step was caused by the pressure regulation. The overshoot was only within a few seconds. The short pulses did not influence the shape of the creep curves, as also published by Yanez et al. [30].

**Figure 3:**
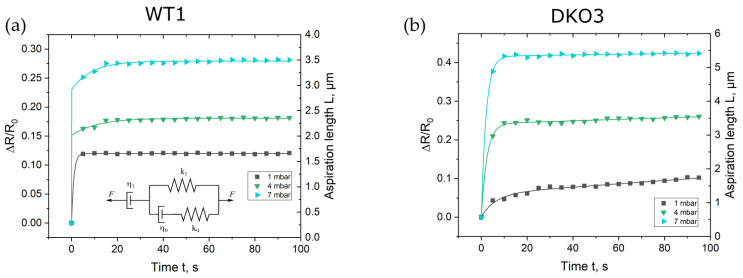
Calculated normalized resistance change ∆*R*⁄*R*_0_ = (*R* −*R*_0_)⁄*R*_0_ at 30 kHz with regard to time for the selected pressure steps for oocyte WT1 (**a**) and oocyte DKO3 (**b**). *R* represents the resistance of the ZP at the rim of the microhole and *R*_0_ is the resistance of the open (not sealed with an oocyte) microhole, respectively. *R*_0_ was determined to be 27.9 Ω and 26.1 Ω for the WT1 and DKO oocytes, respectively. The curves present the aspiration length *L*, which was calculated by fitting the proposed GM model to the data points.

Compared to the gold standard approach in which the Young’s modulus is calculated by the evaluation of the aspirated tissue after relaxation at equilibrium, the focus here was on the analysis of the curve shapes derived directly after the pressure step. As described above, the impedance magnitudes were fitted with the EEC model, from which the suction pressure depending ∆*R*/*R*_0_ was determined in regard to time. In Figure 3, the corresponding data points for the first, fourth, and seventh pressure steps are shown, whereas in Figure S1 in the Supplementary Materials, the plots for the remaining four steps are depicted. Data points were plotted for a time interval of 100 s after each pressure step.

All curve shapes of every investigated oocytes showed the typical behavior of a creep curve. The curves exhibited the well-known shape of a creep curve of a viscoelastic material with its fast elastic deformation (instant elongation) and the following viscous flow. Creep curves can be fitted by equivalent mechanical circuit (EMC) models consisting of dashpots and springs, which can be composed of generalized Maxwell (GM) or Kelvin–Voigt (GKV) EMC models [31]. A dashpot represents viscosity and a spring element Young’s modulus, respectively. Guevorkian et al. and Yanez et al. proposed a modified GM for creep curves obtained with the micropipette aspiration technique, which is shown as an insert in Figure 3 and Figure S1 in the Supplementary Materials [30,32]. The parallel circuit represents the initial elastic deformation. The spring constant *k*_1_ is related to the elasticity of the ZP and therefore to the Young’s modulus *E*, *k*_0_ to the initial jump of the impedance signal and *η*_0_ to the local friction coefficient, respectively. Typical high values of *k*_0_ and low values of *η*_0_ (Table S1) represent the rapid response of ZP’s aspiration to the applied suction pressure in our experiments. The dashpot *η*_1_ in series with the parallel circuit accounts for the viscous dissipation of the aspirating ZP. The correlation of the circuit elements to the creep curve is illustrated in Figure S2 in the Supplementary Materials. Based on this model, a creep curve can analytically be described by Equation (3) [32]

(3) $$L=F\left(\frac{1}{k_{1}}\left(1-\frac{k_{0}}{k_{0}+k_{1}}e^{-t/\tau }\right)+\frac{t}{3\pi^{2}r_{i}\eta_{1}}\right)$$

$$k_{1}=E\pi r_{i}\;F=\pi r_{i}^{2}\Delta p\;\tau =\frac{\eta_{0}\left(k_{1}+k_{0}\right)}{k_{1}k_{0}}$$

where $r_{i}$ is the inner radius of the microhole; *F* is the aspiration force; τ is the retardation time constant.

First, *L* values were calculated for each pressure step according to Equation (2) with the aid of the ∆*R*/*R_0_* values over the whole time period of the pressure step. Then, the *L* values were fitted with the proposed GM model according to Equation (3). The calculations were carried out using Wolfram Mathematica software. For the fittings, the pressure Δ*p*, radius of microhole *r*_i_, and aspiration length *L* were set as constants for all fittings. The Young’s modulus *E*, viscosity *η*_1_, initial jump of the impedance signal *k*_0_, and the local friction coefficient *η*_0_ were free parameters obtained from the fitting. Table S1 in the Supplementary Materials summarizes the fitting parameters for the creep curves of each pressure step of oocytes WT1 and DKO3. After extracting parameters *E* and *η*_1_ from the seven creep curves, their mean values were set as constants. A crosscheck was carried out for all curves to find the minimum deviation. Table S2 in the Supplementary Materials contains the mean values of the fitting parameters for all characterized oocytes. Exemplary fitting curves are depicted as colored lines in Figure 3 and Figure S1 in the Supplementary Materials for the WT1 and DKO3 oocytes. In general, all fitted curves corresponded well to the measured data.

To calculate the Young’s modulus, Equation (3) was inserted into Equation (1). In Table 1, the Young’s moduli of the ZPs of the two characterized oocyte genotypes are summarized and compared to values obtained with the micropipette aspiration technique as well as with the values calculated with our previously published procedure.

Furthermore, the viscosities were calculated from the fitting curves. In the proposed GM model, the fitting parameter η_1_ corresponds to viscosity. Following the model of Guevorkian et al. [32], viscosities were determined to be $6\times 10^{5}\pm 0.8\times 10^{5}$ Pa∙s for the WT and $3.5\times 10^{4}\pm 0.45\times 10^{4}$ Pa∙s for DKO ZPs, respectively.

## 4. Discussion

The objective of this study was to develop and apply an appropriate tool to characterize the viscoelastic properties of the ZP of oocytes. The developed microfluidic chip with its microhole in the cover lid of the microfluidic channel, as shown in Figure 1, represents a significant benefit with regard to the commonly used micropipette aspiration technique. There, the oocyte must be placed manually at the micropipette tip. In contrast to this challenging procedure, the trapping of the oocyte to the microhole is easy to carry out in our MAEIS approach by only placing the cell near the microhole and applying a sufficiently high suction pressure to the microfluidic channel.

The current microfluidic chip design offers the opportunity for multiplexing. Actually, nine micro channels are connected to the pressure regulation unit. This allows for a simultaneous characterization of multiple oocytes. The number of channels can be easily increased. In the current setup, the four wires for the EIS measurements at one microhole were selected by the mini dual in-line package (DIP) switches. In an improved setup, electrodes can be addressed through a chip-based multiplexer. Furthermore, the here utilized bulky impedance spectrometer can also be replaced with a chip-based version. Therefore, the setup could lead to a cheap and small-sized instrument for quantitative oocyte characterization.

In response to a suction pressure step, the obtained curves can be interpreted as the time dependent deformation of the ZP to equilibrium. All creep curve profiles showed nearly the same progression. Similar creep curves were published by Yanez et al. for mouse zygotes [30]. Therefore, we can conclude that ZP expresses a linear viscoelastic behavior.

As shown in Table 1, the Young’s moduli of ZPs, which were calculated on the basis of creep curves, showed a high concordance in comparison to those values obtained with the evaluation of the saturation values of the impedance after each suction pressure step, as published by Cao et al. [24]. It can be concluded that with the same setup and measurement routine, the Young’s modulus can be calculated with two different evaluation routines. The Young’s modulus of the WT oocyte was significantly smaller than the value obtained with the micropipette aspiration techniques: when looking to the ratio of the aspiration length to the inner radius of the microhole *L*/*r*_i_, the groups working with the standard micropipette aspiration technique published ratios 0.2 < *L*/*r*_i_ < 1.6. In our measurements, the factor was calculated to be much lower at 0.045 < *L*/*r*_i_ < 0.14. It is most likely that we only aspirated a very small amount at the surface of the hydrogel-like ZP, so we did not measure the bulk properties. Thus, the Young’s modulus obtained with our MAEIS setup was estimated to be lower than the value that would be obtained for a larger amount of ZP sucked into the hole. We therefore developed a surface-sensitive system. Nevertheless, relative values of the Young’s moduli bear more meaningful information than absolute values when the ZP’s modification is measured over time to find the window of highest fertility.

It is worth mentioning that the analysis of the creep curves via fitting the EMC to the experimental data additionally allows for the calculation of the viscosity of the ZP, which is not possible when only the saturation values of the electrical impedances during the pressure steps are evaluated. To the best knowledge of the authors, this is the first time that absolute values of viscosities have been published for the ZP of mouse oocytes. Kim et al. published only relative values [33], whereas Yanez et al. presented only a value for the dashpot *η*_1_ for mouse zygotes and not for the viscosity [30].

It has to be noted that microfluidic devices have already been developed that enable the simultaneous characterization of both the mechanical and electrical properties of single cells [34,35,36,37]. In these devices, cells were trapped at constrictions in microfluidic channels. The Young’s moduli were calculated by optically measuring the aspiration length changes induced through a variation in suction pressure. The capacitance of the whole cell was determined from the EIS measurements. However, in these approaches, the electrodes for EIS were located in reservoirs on both sides of the microfluidic channel with electric field lines penetrating the whole cell. In comparison, the microhole in our MAEIS setup was localized between ring-shaped electrodes. Here, the electrical field lines were forced through the microhole and concentrated at the rim of the microhole [24]. By aspirating only the ZP into the microhole, the mechanical property of the ZP alone instead of the whole cell was characterized.

## 5. Conclusions

Using the MAEIS setup, the transient electrical impedance spectra measured were caused by the aspiration of the ZP of a mouse oocyte into a microhole connected to a microfluidic suction channel. The curve progression was correlated to a typical viscoelastic creep behavior and fitted by an appropriate equivalent mechanical model. In combination with a mathematical model, which correlates the aspiration with the impedance change, the Young’s moduli and, for the first time, the absolute viscosity values of the ZPs of two mouse genotypes were successfully calculated. The work showed that with the same setup and measurement routine, the obtained impedance spectra could be analyzed by two different approaches: the one already published by Cao et al. [24], which evaluates the heights of the impedance signals with regard to the pressure step, and the method described in this work by evaluating the creep curves.

This study showed that our approach offers several advantages. The chip-based microfluidic aspiration technique in combination with the electrical impedance spectroscopy opens the possibility of studying the process of ZP hardening with high spatial and temporal resolution. With the evaluation of the creep curves, the Young’s modulus as well as the viscosity can be determined. The setup can be improved to study several cells simultaneously. The work demonstrates a step forward to a user-friendly portable point-of-use tool to characterize the rheological properties of any kind of individual cell.

## Figures and Tables

**Table 1:** Calculated mean values of the Young’s moduli of ZP of mouse oocytes in the metaphase II (MII) stage. The errors represent standard deviations.

Cell Type (Maturation Stage)	Technique	Young’s Modulus (kPa)	Reference
Oocyte (wild type, WT)	Micropipette aspiration	10.9 ± 1.4	[20]
Oocyte (wild type, WT)	Micropipette aspiration	11.8	[19]
Oocyte (wild type, WT)	Micropipette aspiration	14.3 ± 2.1	[22]
Oocyte (wild type, WT)	Micropipette aspiration	8.2 ± 1.2	[21]
Oocyte (wild type, WT)	MAEIS	3.58 ± 0.63	[24]
Oocyte (wild type, WT)	MAEIS (creep)	3.3 ± 0.5	Current study
Oocyte (Fetuin-B/ovastacin dd, DKO)	MAEIS	1.19 ± 0.30	[24]
Oocyte (Fetuin-B/ovastacin dd, DKO)	MAEIS (creep)	1.1 ± 0.2	Current study

dd: double deficient; WT: wild type; DKO: fetuin-B/ovastacin double deficient; MAEIS: microfluidic aspiration-assisted electrical impedance spectroscopy.

## Data Availability

The data presented in this study are available on request from the corresponding author.

## Supplementary Materials

The following supporting information can be downloaded at: https://www.mdpi.com/article/10.3390/bios13040442/s1, Figure S1. Calculated normalized resistance change ∆*R*⁄*R*_0_ = (*R* − *R*_0_)⁄*R*_0_ at 30 kHz in regard to time for selected pressure steps for oocyte WT1 (**a**) and oocyte DKO3 (**b**).; Figure S2. Schematic of the overlap of an ideal normalized resistance change ∆*R*⁄*R*_0_ (black thick dashed line) and an ideal creep curve (red) and for a constantly applied suction pressure step.; Table S1. Fitting parameters to Figure 3 and Figure S1 according to the Equation (3) in the main text.; Table S2. Summarized fitting data for the four wild type (WT) and four fetuin-B ovastacin double deficient (DKO) MII oocytes.
