# Supplementary material for: Viscoelastic Properties of Zona Pellucida of Oocytes Characterized by Transient Electrical Impedance Spectroscopy
Source: Biosensors (Basel). 2023 Mar 30;13(4):442. doi: 10.3390/bios13040442 (PMC10136587; doi:10.3390/bios13040442)
Supplement: Supplementary file 1 [file biosensors-13-00442-s001.zip › biosensors-2209639-supplementary.pdf]

biosensors

Supporting Information

Viscoelastic Properties of Zona Pellucida of Oocytes Characterized by Transient Electrical Impedance Spectroscopy

Danyil Azarkh [1,†], Yuan Cao [1,†], Julia Floehr [2] and Uwe Schnakenberg [1,*]

[1] Institute of Materials in Electrical Engineering 1, RWTH Aachen University, Sommerfeldstraße 24, 52074 Aachen, Germany; azarkh@iwe1.rwth-aachen.de; yuan.cao@iwe1.rwth-aachen.de
[2] Helmholtz-Institute for Biomedical Engineering, Biointerface Laboratory, RWTH Aachen University, Pauwelsstraße 30, 52074 Aachen, Germany; julia.floehr@rwth-aachen.de
† These authors contribute equally to the work.
* Correspondence: schnakenberg@iwe1.rwth-aachen.de.

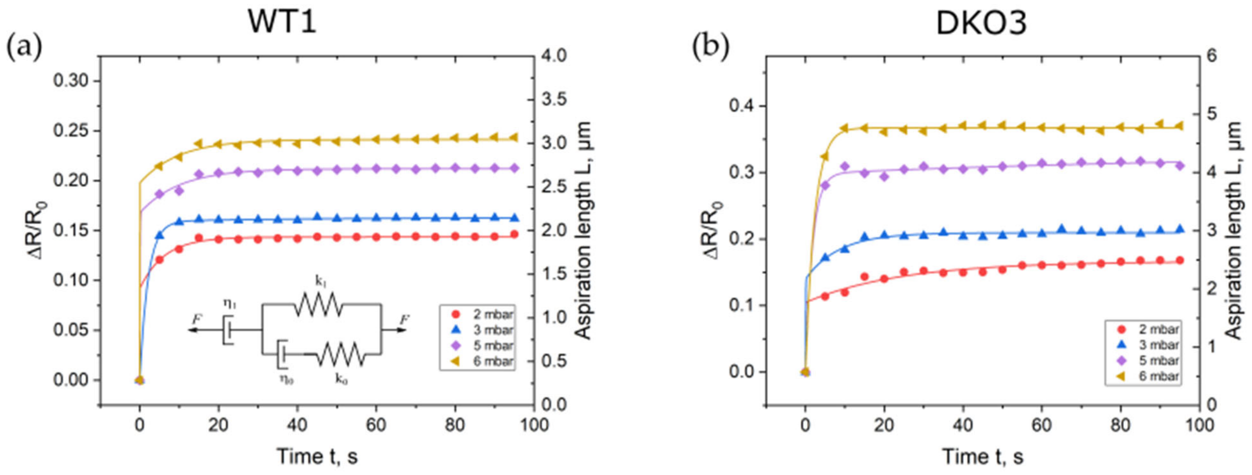

Figure S1. Calculated normalized resistance change $\Delta R/R_0 = (R-R_0)/R_0$ at 30 kHz in regard to time for selected pressure steps for oocyte WT1 (**a**) and oocyte DKO3 (**b**). R represents the resistance of the ZP at the rim of the micro hole and R_0 the resistance of the open (not sealed with an oocyte) micro hole, respectively. For oocyte WT1, R_0 was determined to be 27.9 kΩ, whereas for DKO oocyte, R_0 was calculated to 26.1 kΩ, respectively. The aspiration lengths L, calculated from fitting the EMC model, respectively Equation (3) in the main text, to the data points, are shown as lines.

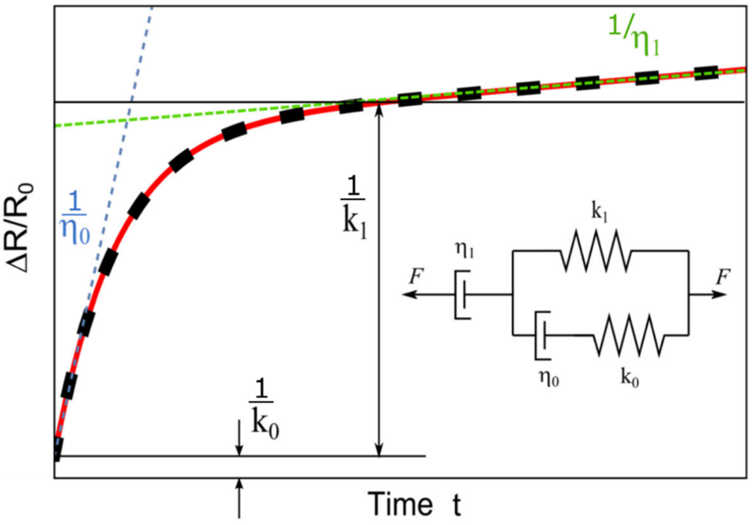

Figure S2. Schematic of the overlap of an ideal normalized resistance change $\Delta R/R_0$ (black thick dashed line) and an ideal creep curve (red) and for a constantly applied suction pressure step. In our proposed calculation procedure, values of the aspiration length L were calculated according to Equation (2) presented in the manuscript with the aid of the $\Delta R/R_0$ values. Then, the L values were fitted with the proposed GM model shown in the insert, respectively Equation (3) in the manuscript. The red line represents the fitting curve. The GM, as proposed by Guevorkian et al. as well as by Yanez et al. [1,2], consists of four elements, two springs k_0 and k_1 and two dashpots η_0 and η_1, respectively. As depicted, the slope of blue dashed line in the ideal creep curve represents the contribution of the local friction coefficient η_0, the slope of the green dashed line the input of the viscosity η_1 of ZP, k_0 the contribution of initial jump of aspiration length, and k_1 the Young's modulus of the ZP, respectively.

Equation (S1), definition of Function *C*

According to Reference [3], C is a function of the dimensionless ZP shell thickness $h^* = h/r_i$ and can be approximated by the following form

$$C(h^*_{ZP}) = \begin{cases} \dfrac{a + c\ln(h^*) + e\ln^2(h^*) + g\ln^3(h^*) + i\ln^4(h^*)}{1 + b\ln(h^*) + d\ln^2(h^*) + f\ln^3(h^*) + h\ln^4(h^*) + j\ln^5(h^*)} & for\ 0.1 \le h^* \le 50 \\ 0.64395655 & for\ 50 \le h^* \end{cases} \quad \text{(S1)}$$

with a= 1.070275412, b= 0.592405186, c= −0.44373783, d= 0.126723221, e= 0.721290633, f= 0.074985305, g= −0.14390482, h= 0.027220129, i= 0.040156098, j= 0.00132358.

Table S1. Fitting parameters to Figure 3 and Figure S1 according to the Equation (3) in the main text.

Oocyte Type	Pressure, hPa	$k_0 \times 10^{14}$, Pa	$E \times 10^3$, Pa	$\eta_0 \times 10^{-12}$, Pa s	$\eta_1 \times 10^5$, Pa s	Coefficient of determination R^2
WT1	1	2.1 ± 1.8	3.3 ± 0.23	7.8 ± 5.5	5.9 ± 0.4	0.998
WT1	2	1.8 ± 1.5	3.5 ± 0.25	8.5 ± 3.9	5.8 ± 0.3	0.989
WT1	3	1.9 ± 1.1	3.8 ± 0.18	9.2 ± 3.3	6.2 ± 0.4	0.996
WT1	4	2.5 ± 1.9	3.3 ± 0.2	9.6 ± 6.4	6.4 ± 0.8	0.988
WT1	5	2.8 ± 1.2	3.6 ± 0.12	9.4 ± 6.6	6.8 ± 0.6	0.995
WT1	6	2.4 ± 2.2	3.8 ± 0.15	8.5 ± 6.3	6.1 ± 0.6	0.997
WT1	7	2.5 ± 1.4	3.7 ± 0.1	8.4 ± 4.5	5.9 ± 0.2	0.995
DKO3	1	3.3 ± 1.7	1 ± 0.17	4.9 ± 4.5	0.5 ± 0.1	0.989
DKO3	2	6 ± 4.2	0.8 ± 0.12	5.5 ± 5.1	0.3 ± 0.04	0.987
DKO3	3	4.2 ± 3.1	0.9 ± 0.24	5.7 ± 4.7	0.3 ± 0.09	0.997
DKO3	4	2.4 ± 1.9	1.2 ± 0.21	5.5 ± 4.4	0.4 ± 0.12	0.994
DKO3	5	5.5 ± 2.4	1 ± 0.18	5.9 ± 4.9	0.3 ± 0.07	0.996
DKO3	6	5.3 ± 3.8	1.8 ± 0.28	5.2 ± 3.1	0.1 ± 0.05	0.997
DKO3	7	4.3 ± 3.5	1.4 ± 0.25	5.8 ± 5.2	0.2 ± 0.03	0.998

Table S2. Summarized fitting data for the four wild type (WT) and four fetuin-B ovastacin double deficient (DKO) MII oocytes.

Oocyte Type	$k_0 \times 10^{14}$, Pa	$E \times 10^3$, Pa	$\eta_0 \times 10^{-12}$, Pa s	$\eta_1 \times 10^5$, Pa s
WT1	2.3 ± 1.3	3.6 ± 0.17	8.7 ± 6.5	6.1 ± 0.26
WT2	1.3 ± 1.24	3.5 ± 0.36	9.6 ± 5.53	5.9 ± 0.91
WT3	1.6 ± 1.46	3.3 ± 0.23	9.5 ± 6.74	5.8 ± 0.6
WT4	2.2 ± 1.19	2.8 ± 0.46	9.3 ± 6.62	6.2 ± 0.92
DKO1	4.5 ± 2.19	0.9 ± 0.15	5.3 ± 4.6	0.41 ± 0.15
DKO2	4.1 ± 2.46	1.1 ± 0.33	5.4 ± 5.24	0.32 ± 0.1
DKO3	4.2 ± 2.01	1.2 ± 0.26	5.5 ± 4.25	0.29 ± 0.08
DKO4	3.8 ± 2.72	1.2 ± 0.1	5.9 ± 4.22	0.38 ± 0.13

References

1 Guevorkian, K.; Colbert, M.J.; Durth, M.; Dufour, S.; Brochard-Wyart, F. Aspiration of biological viscoelastic drops. *Phys Rev Lett* **2010**, *104*, 218101, doi:10.1103/PhysRevLett.104.218101.

2 Yanez, L.Z.; Han, J.; Behr, B.B.; Pera, R.A.R.; Camarillo, D.B. Human oocyte developmental potential is predicted by mechanical properties within hours after fertilization. *Nat Commun* **2016**, *7*, 10809, doi:10.1038/ncomms10809.

3 Alexopoulos, L.G.; Haider, M.A.; Vail, T.P.; Guilak, F. Alterations in the mechanical properties of the human chondrocyte pericellular matrix with osteoarthritis. *J Biomech Eng* **2003**, *125*, 323-333, doi:10.1115/1.1579047.
